# AI Based Monitoring of Different Risk Levels in COVID-19 Context

**DOI:** 10.3390/s22010298

**Published:** 2021-12-31

**Authors:** César Melo, Sandra Dixe, Jaime C. Fonseca, António H. J. Moreira, João Borges

**Affiliations:** 1 Engineering School, University of Minho, 4800-058 Guimarães, Portugal; 2 Algoritmi Center, University of Minho, 4800-058 Guimarães, Portugal; jaime@dei.uminho.pt (J.C.F.); jpbsilva@algoritmi.uminho.pt (J.B.); 3 2Ai, IPCA, School of Technology, 4750-810 Barcelos, Portugal; amoreira@ipca.pt; 4 Polytechnic Institute of Cávado and Ave, 4750-810 Barcelos, Portugal

**Keywords:** COVID-19, deep learning, supervised learning, object detection, keypoint detection

## Abstract

COVID-19 was responsible for devastating social, economic, and political effects all over the world. Although the health authorities imposed restrictions provided relief and assisted with trying to return society to normal life, it is imperative to monitor people’s behavior and risk factors to keep virus transmission levels as low as possible. This article focuses on the application of deep learning algorithms to detect the presence of masks on people in public spaces (using RGB cameras), as well as the detection of the caruncle in the human eye area to make an accurate measurement of body temperature (using thermal cameras). For this task, synthetic data generation techniques were used to create hybrid datasets from public ones to train state-of-the-art algorithms, such as YOLOv5 object detector and a keypoint detector based on Resnet-50. For RGB mask detection, YOLOv5 achieved an average precision of 82.4%. For thermal masks, glasses, and caruncle detection, YOLOv5 and keypoint detector achieved an average precision of 96.65% and 78.7%, respectively. Moreover, RGB and thermal datasets were made publicly available.

## 1. Introduction

In December 2019, an outbreak of pneumonia with unknown origins was reported in Wuhan, China. After conducting several tests around the associated virus, it was concluded to be a new variant of the existing coronavirus, associated with SARS-CoV. On 12 March 2020, the WHO declared a state of global emergency, considering it a pandemic, after nearly 125,000 cases were reported to be spread across more than 118 countries at that point. Since then, strict measures were implemented worldwide to contain the spread of the virus and reduce the chains of contagion, due to the virus’ high level of transmissibility and inherently devastating effects, especially in people with chronic diseases, with weakened immune systems, and with those of older age. These measures severely affected all sectors, from the closure of the overwhelming majority of public establishments to bans on movement on public roads. The main symptoms of this disease are fever, cough, headaches, fatigue, and loss of taste, and its transmission through droplets released by the nose and mouth required rules of physical distancing and the mandatory use of masks in all activities that imply direct or indirect contact [1].

In this way, the need to develop advanced systems capable of monitoring people’s behavior in an optimized way, especially in places that generate large concentrations of people in small areas, thus reducing as much as possible the spread of the virus within the community.

With the easing of restrictions, the levels of mobility and concentration of people, especially in public spaces and shopping areas, began to gradually increase again. However, the persistent presence of the virus means that behavior must still be moderate and adopt every precaution so that the number of infections and new infections remains at increasingly lower values to return normality in a manner as accelerated as possible. The fact that many people are asymptomatic to the disease also contributes to careless attitudes and negligent behavior, mainly associated with not wearing a mask. These behaviors and risk factors are imperative to monitor. Since this type of management is quite complicated to conduct in terms of human resources (e.g., at the entrances to shopping areas, where there are multiple entrance points and a large influx of people simultaneously), it is necessary to adopt methodologies that allow this monitoring to be performed in a more simple and optimized manner.

This article involves the presentation of the study and implementation of algorithms that allow, in real-time, the identification of risk factors and behaviors, such as the detection of the presence or absence of masks in people, as well as the precise measurement of their body temperature, to identify risk factors regarding possible cases of virus presence. This paper can be divided into two distinct modules: (A) detection of the presence or absence of masks on people in places where its use is mandatory, and secondly, (B) a punctual temperature measurement to detect situations where people are in a feverish state, one that represents a key symptom of the SARS-CoV-2 virus. Moreover, such algorithmic development is suitable to be implemented in an integrated system that allows to deploy a product in the market for monitoring purposes.

The main contributions of the paper are as follows:

1. A methodology for the generation of hybrid datasets with added masks on top of real samples from public datasets (Section 3.1.1);
2. An RGB dataset with added synthetic masks, on top of public datasets. MoLa RGB CovSurv [2] was made publicly available.
3. An IR dataset with information on the presence of the caruncle, masks, and glasses. MoLa IR CovSurv [3] was made publicly available.
4. State-of-the-art object detectors and keypoint face detectors were trained and evaluated, using a hyperparameter genetic search algorithm, i.e., Evolve. Considering the highest precision and lowest computational requirements, two models were selected. YOLOv5 small was the best choice for the RGB and IR mask and glasses detection. Moreover, a keypoint detector with a Resnet-50 backbone was selected for the caruncle detection in IR images.

**Figure 1:**
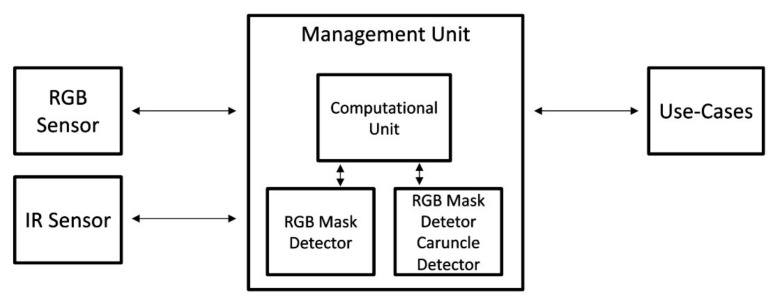
Architecture description of purposed solution.

Using these algorithms, we can implement them in an embedded system, and using the RGB cameras, we can install this as a monitoring system to assist with controlling the entrance of crowded establishments. Furthermore, it replaces the in-person task of measuring body temperature. The architecture description of the proposed solution can be consulted in Figure 1.

The paper is organized as follows. Initially, the state-of-the-art is presented regarding deep-learning-based algorithmic solutions for the use-case at hand (i.e., RGB mask detection and IR keypoint detection).

In the implementation section, for the RGB mask detection, a public dataset collection was made. Moreover, due to the lack of contextualized samples, a synthetic data generation toolchain was developed to generate the MoLa RGB CovSurv dataset [2].

For the IR algorithmic development, the same procedure was used, and publicly available datasets were used to create a pool of samples with extra label information (i.e., caruncle, mask, and glasses position). Moreover, a new MoLa IR CovSurv dataset [3] was formed.

Several evaluations were performed for RGB and IR detection use-cases using the generated datasets, where YOLOv5 was used as the main object detector, and a keypoint detector, based on the Resnet-50 backbone, was used for the caruncle detection.

Finally, results are presented and discussed, making it possible to select the best algorithms with the highest precision and lowest computational requirements.

**Figure 2:**
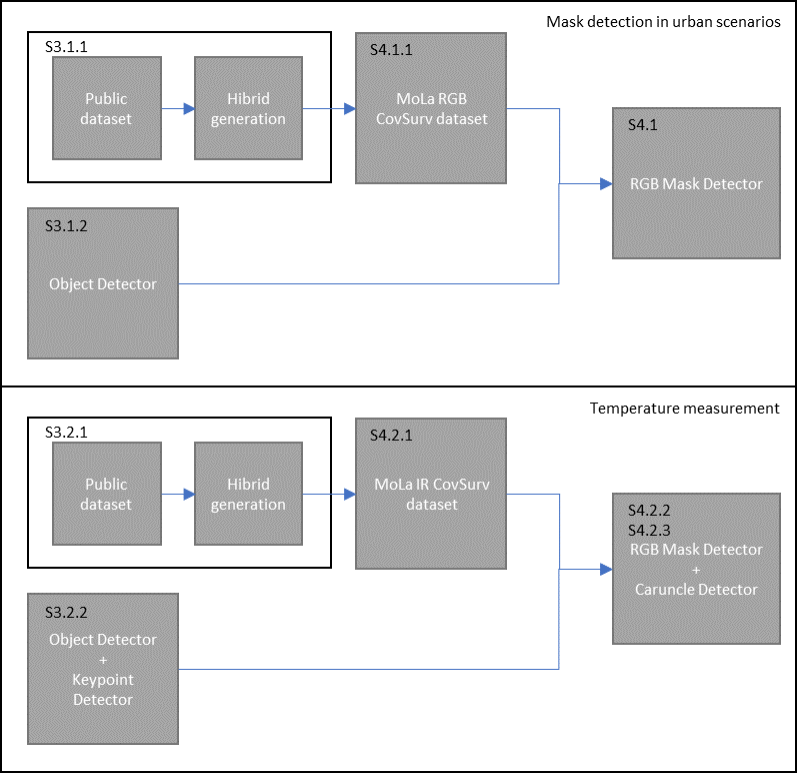
Two development pipelines are presented: (1) mask detection in urban surveillance; (2) temperature measurement. For each pipeline, 3 identical steps are made: (1) a toolchain for data generation is implemented (S3.1.1 and S3.2.1); (2) models are selected (S3.1.2 and S3.2.2); (3) datasets are generated (S4.1.1 and S4.2.1) to evaluate each model (S4.1, S4.2.2, and S4.2.3).

Figure 2 summarizes the entire development pipeline of this article.

## 2. Related Work

Human mask detection in a surveillance scenario requires an approach similar to the ones used in object detection methodologies. There are several studies focused on object detection, which can be applied to various topics, and which can be an approach to consider in the task of mask detection. The authors [4,5,6] developed the R-CNN family of algorithms to detect different regions of interest in the image while using a CNN to classify the presence of the object in that region. More recently, the YOLO [7] object detection family presented as YOLOv2 [8], YOLOv3 [9], YOLOv4 [10], and YOLOv5 [11], provide a more accurate and faster method compared to the R-CNN family. Most recently, several object detection algorithms were used for the sole purpose of mask detection in a COVID-19 context. Jiang et al. [12] proposed a one-stage detector, achieving state-of-the-art results on a public face mask dataset. In the same context, Loey et al. [13] used YOLOv2 with a Resnet-50 backbone with two publicly available medical masks dataset, reaching an average precision of 81%. Alternatively, the authors [14] used a single-shot detector with a MobileNetv2 backbone for the sole purpose of detecting masks in a surveillance scenario. Moreover, public datasets with real and synthetic samples were used for the algorithmic development, allowing to achieve 92.64% accuracy, with 64ms of inference time.

For the detection of facial points, an important requirement for the detection of the caruncle location in human faces, state-of-the-art algorithms were developed. The first efficient algorithm for face detection in images was presented in 2001 by [15]. Later, in 2015, the authors [16] presented a cascaded CNN model, i.e., using 3 distinct CNNs (12-net, 24-net, and 48-net), in which a gradual analysis of the image is performed, and initially, several small boxes are generated, which refer to certain facial elements; throughout the process, dimensional adjustments and calibrations are made until the face is identified as a whole. Sun et al. [17] presented an algorithm consisting of three levels of CNNs in cascade form for the detection of the five main facial points: Left-Eye Center (LE), Right-Eye Center (RE), Nose Tip (N), Left-Mouth Corner (LM) and Right-Mouth Corner (RM). It is a supervised approach, and when the bounding box of a face is provided, the location of the respective points is predicted. Haavisto et al. [18] presents a DBN-based algorithm to identify 15 facial points based on grayscale images. Longpre et al. [19] presented an approach to predict facial features in grayscale images. This algorithm consists of a mixture of convolutional layers based on the architectures of CNNs LeNet and VGG. Upon reception of an image, the goal is to return the coordinates (x,y) of 30 facial points. Agarwal et al. [20] presented NaimishNet, an adaptation of LeNet architecture architecture for identifying facial features.

Several studies were already developed to monitor risk behavior in an attempt to mitigate the spread of COVID-19.

The author [21] proposed a monitoring and warning approach to respect social distancing (SD), relying on vision systems, and it was effective at preventing the spread of COVID-19 infectious disease. In this study, a real-time, vision-based system that can detect SD violations and send nonintrusive audio-visual cues using recent DL models is presented. A critical value of social density was defined, and they showed that the probability of occurrence of SD violation can be kept close to zero if the pedestrian density is kept below this value. The proposed system is also ethically fair: it does not record data or target individuals, and no human supervisor is present during operation. The proposed system was evaluated on real-world datasets.

The author [22] proposed a detection and diagnosis system using IoT-based smart glasses that can automatically and quickly detect COVID-19 from thermal images. The proposed design can perform face detection in case of suspected COVID-19 among crowds that have high body temperatures. The design will add information on the visited location of the suspected virus carriers through Google Location History (GLH) to provide reliable data on the detection process.

The authors [23,24] evaluated the probability of the COVID-19 disease through sound analysis. Ref. [23] proposed the study of voice (speech) signal processing in the process of screening and early diagnosis of the COVID-19 virus, using Recurrent Neural Network (RNN), and more specifically, its well-known architecture, Long Short-Term Memory (LSTM), to analyze the acoustic characteristics of cough, breath, and voice of patients. The presented study shows a low accuracy in the voice test compared to that of the cough and breath sound samples. However, they highlight the possibility of increasing the accuracy of voice testing by expanding the dataset and targeting a larger group of healthy and infected people. Ref. [24] proposes a study that analyses cough sound. They present a reliable tool that can differentiate between different respiratory diseases, which is very relevant in the COVID-19 context.

The authors [25,26] present DL approaches for detecting or not face masks on individuals. Ref. [25] proposes a system that restricts the growth of COVID-19 by tracking people not wearing a face mask in a smart city network where all public places are monitored by Closed Circuit Television (CCTV) cameras. While a person without a mask is detected, the corresponding authority is informed through the city network. It uses a DL architecture trained on a dataset consisting of images of people with and without masks collected from various sources. The trained architecture achieved 98.7% accuracy in distinguishing people with and without face masks using previously unseen test data. Ref. [26] proposes the implementation of a facial mask and social distancing detection model as an embedded vision system. The pretrained models such as MobileNet, ResNet classifier, and VGG are used in our context. People violating social distancing or not wearing masks were detected. After the implementation and deployment of the models, the selected one achieved a 100% confidence index.

## 3. Implementation

### 3.1. Urban Mask Detection

#### 3.1.1. Synthetic Dataset Generation

**Figure 3:**
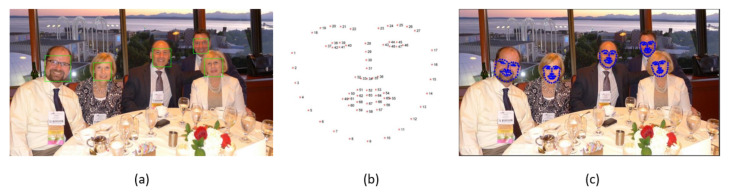
(**a**) Output of Get_Frontal_Face_Detected method of Dlib library. (**b**) Face location of each of shape_predictor_68_face_landmarks. (**c**) Output of pretrained model application shape_predictor_68_face_landmarks.

Since the amount of data used is also a very relevant factor for obtaining reliable and robust models, the need to develop a tool capable of generating synthetic images as a way to increase the available data arose. This tool was developed so that a wide variety of masks can be applied to public datasets, taking into account the position and orientation of faces, mask placement zone, and mask usage probability (as shown in Figure 3). For the generation of this synthetic dataset, it was decided to put synthetic masks on images of public datasets; thus, to perform this task, the first step is to find the faces of people in the images, and for this we used the method present in the open source library Dlib [27]. This method corresponds to a pretrained model based on HOG and SVM, which identifies faces in images, returning an object for each face detected. This object is of the type “rectangles”, formed by two tuples representing, respectively, the coordinates of the upper-left and the lower-right corner points, which allow the formation of a rectangle around the detected face. Next, a function is applied that converts these two tuples into a bounding box. After extracting the bounding boxes associated with the faces present in the image, another pretrained method is used from the dlib library. Given the input image and the corresponding Region of Interest (ROI) (i.e., face detected by the previous method), the method tries to locate the face keypoints of interest within that region. In this tool, the detector estimates 68 2D points (x,y) associated with the other facial regions.

**Figure 4:**
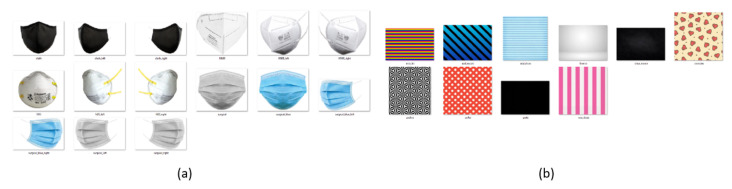
(**a**) Mask templates used when applying them synthetically to detected faces. (**b**) Set of examples among many mask textures applied with this tool.

After the identification process of faces and respective facial keypoints, if more than 3 faces are identified in the image, 80% of the faces are randomly selected to be processed with the application of a synthetic mask, while the remaining 20% of the faces will remain unmasked. This methodology allows for an increase in the robustness of the algorithms to be trained, since, in this way, the final dataset will not be formed only by images with or without masks. For each of the faces to be masked, the type of mask is randomly selected, whether or not a texture will be applied and, if so, which texture will be used. The models of masks and some of the textures used can be seen in Figure 4a,b.

Since not all the faces of the other datasets are in a frontal position in relation to the camera that captured them, affine transformations are performed on the other facial points obtained in the second step to understand which portion of the face is visible. For this reason, and as shown in Figure 4a, different perspectives are available for each mask model, according to the facial visibility. The samples in which this tool was used belong to the already existing datasets listed in the Table 1. The use of different datasets, in addition to increasing the number of samples, allows to enhance the algorithms to be trained, since there are samples with different quality levels, occlusion, luminosity, background, and number of people.

**Figure 5:**
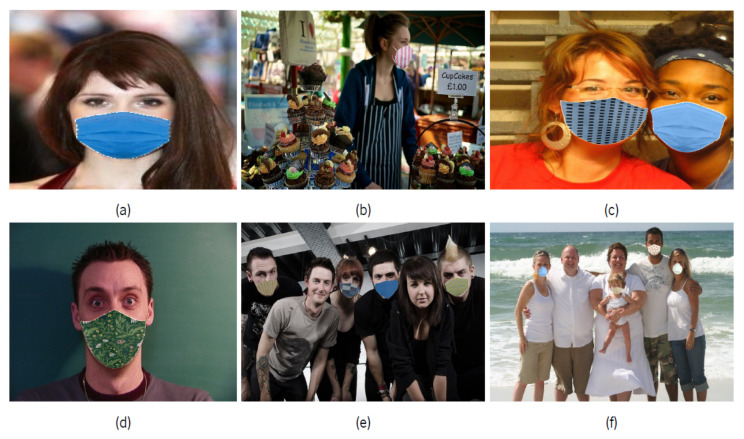
Examples of samples subjected to application of synthetic masks from MobileFaceNet tool. (**a**) Dataset Celeba. (**b**) Dataset Coco. (**c**) Dataset Helen. (**d**) Dataset IMM. (**e**) Dataset Wider. (**f**) Dataset Group Images.

After performing some tests of the tool on the different samples present in the datasets listed in Table 1, it was concluded that the method associated with the dlib library for detecting facial points was not very effective when the faces were not at a relatively frontal angle; the identification of the faces happened, but they were considered as if they were at a frontal angle, which led to poor applications of the synthetic masks. An individual analysis was made of all the samples used to discard those in which the tool did not work as expected. For these incorrect samples, the respective annotations of the other datasets were used, in the cases where they were provided, to obtain the exact facial points for the correct application of the synthetic mask. In situations where the samples were not accompanied by annotations, another pretrained model was used, called MobileFaceNet [34], capable of predicting more accurately the same 68 points associated with each face, even if they are not visible in the image. Some final results obtained from the use of the tool can be verified in the Figure 5.

For this dataset, two classes were considered, “With Mask” and “Without Mask”, to which IDs were assigned “0” and “1”, respectively. As the object to be identified is always the face of a person, regardless of the presence or absence of mask, the labels associated to each image were based on the information provided by the method get_frontal_face_detector applied in the first stage of the tool for applying synthetic masks, which is responsible for identifying the faces present in a sample from the return of the coordinates delimiting each of the objects found. Thus, it was only necessary to normalize this data according to the dimensions of each image. Finally, the MoLa RGB CovSurv dataset was generated, and made publicly available [2].

The number of labels associated with class “0” (face with mask) is approximately 55,000 and class “1” (face without mask) is 20,000. These labels are used for training the selected algorithms. This imbalance is due to the fact that with the tool presented in Section 3.1.2, masking was applied to 80% of the identified faces in each sample with more than three identified in each sample, since, in real situations, the tendency is the presence of a large majority of masks.

#### 3.1.2. Model

To perform the mask detection task the YOLOv5 family will be used, specifically the small, medium, large, and extra-large models, which differ in the depth of their layers, real-time performance, and detection accuracy. Input image resolution was fixed at a 512 × 512, with two classes output. Anchor boxes will be calculated automatically for the training dataset.

### 3.2. IR Temperature Detection

#### 3.2.1. Dataset Generation

**Figure 6:**
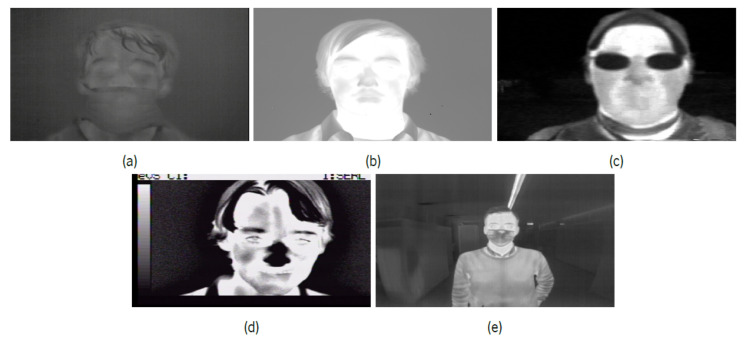
Sample examples of other thermographic datasets used for facial point detection task. (**a**) IIITD In and Beyond Visible Spectrum Disguise Database. (**b**) UL-FMTV Database. (**c**) Terravic Facial Infrared Database. (**d**) IRIS Database. (**e**) Sample collected in laboratory.

Another risk factor that may reflect the presence of the SARS-CoV2 virus is high body temperature, which usually indicates a feverish state. Similarly to the previous chapter, the need arises to collect samples that will be the base for training algorithms capable of identifying, in a thermographic context, the presence of masks, goggles, and facial areas where temperature measurement is carried out in a more reliable way. In this case, it corresponds to the tear area present in each eye of the human being (more specifically, the caruncle area [35]). These samples were obtained from public datasets, and also from samples generated in laboratory. The latter were based on the availability of 30 volunteers to perform a series of recordings in different scenarios. These recordings, using a thermographic camera (FLIR ADK [36]), consisted of the continuous movement of approaching the camera up to a 30 cm distance, followed by the approximation of the face, as a way of making the areas associated with the caruncle visible, for later analysis and creation of labels for algorithm training. In Table 2 are described the existing datasets formed by the thermographic images used in the training of the selected algorithms, and that represent a large portion of the final dataset generated. In Figure 6, it is possible to see some samples of these same datasets.

For the mask and glasses detection component, the labels go through only the location of the face in the image, whose classes to be identified by the selected object detection algorithms are presented in Table 3. For the detection of the facial keypoints of interest, the labels also included the identification of the face in each sample, with the addition of the location of the two points associated with the caruncle of both eyes.

**Figure 7:**
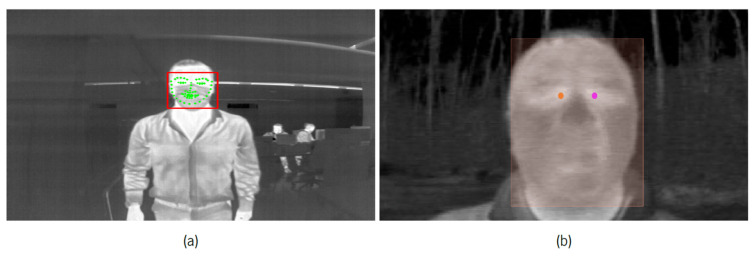
Examples of samples where semiautomatic label technique was applied. (**a**) Automatic Labeling. Red rectangle refers to bounding box of identified face, and green dots to respective facial marks. (**b**) Manual labeling. Orange rectangle is associated to identified face, while orange and violet dots refer, respectively, to points that identify right- and left-caruncles.

**Figure 8:**
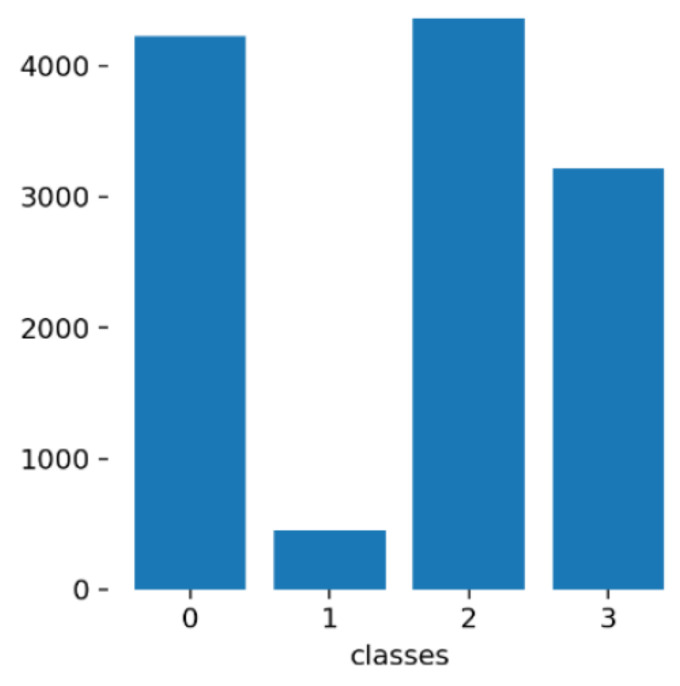
Number of labels associated to different classes existing in mask and glasses detection task. Different classes can be consulted in Table 3.

The necessary labels were generated in a semiautomatic labeling process. In the first stage, all the samples that make up the thermographic dataset were subjected to passing through the pretrained models that constitute the first two steps used for the development of the tool responsible for applying synthetic masks, get_frontal_face_detector and shape_predictor_68_face_landmarks, whose functions include the identification of faces and the location of the 68 points associated with the facial regions. As we are dealing with thermographic images and some of them have masks and/or glasses present, these models presented certain difficulties in identifying the desired information in most of the samples constituting the dataset. Thus, for the samples with satisfactory results, the information returned by the models was converted to the formats used by the different algorithms to associate the labels to the respective images. In situations where the results did not meet expectations, manual labeling was performed using the online tool V7Darwin [41] and the labels to be identified in each image were generated one by one. Figure 7 shows two examples where it is possible to consult the labels obtained automatically and manually. With attention to Figure 7a, although the facial points associated with the mandible and mouth region were poorly identified since a mask is present, the bounding box of the face present as well as the points of interest (left- and right-caruncles) were well identified. In this case, this information was taken into consideration for label formation. Finally, the MoLa IR CovSurv dataset was generated and made publicly available [3]. Figure 8 shows the number of samples that make up the generated dataset, associated with each of the classes to be identified. The great unbalance between class 1, associated with people wearing a mask and wearing glasses, in relation to the other classes, is due to the fact that both in the existing datasets collected, as well as in the people who volunteered to make laboratory recordings, the presence of glasses was quite scarce. Classes 0 and 1 come essentially from the recordings made, where the presence of mask predominates, while classes 2 and 3 belong mostly to the datasets presented in Table 2.

#### 3.2.2. Model

For the detection of masks and glasses in thermographic samples, object detection models are highly contextualized. As such, YOLOv5 was selected for the evaluations, with an input image resolution fixed at 512 × 512, and four classes output. For the caruncle detection a keypoint detector, Ref. [42] was selected, with different backbones available (i.e., Resnet-# and HrNetv2_w#), input image resolution was also fixed at 512 × 512, with two heatmaps output.

## 4. Experiments and Results

The objective of this section is to evaluate the algorithms used for the detection tasks we have set ourselves. The algorithms trained for these tasks are the variants of the YOLOv5 architecture (Section 3.1.2) and the keypoint detection algorithms (Section 3.2.2), whose backbones correspond to architectures of the CNNs Resnet and HRNetv2 families. All these tests were performed on a server with an Intel(R) Xeon(R) Gold 6140 CPU 2.30Ghz processor, 128GB RAM, and NVIDIA Tesla V100-PCIE-16GB computing GPU.

### 4.1. RGB Detection

#### 4.1.1. Dataset

For the RGB detection evaluations, MoLa RGB CovSurv dataset was used. Table 4 shows the description of each subset of the dataset used for mask detection.

The approach presented in Table 4 is unbalanced in quantitative terms and is justified by the fact that the training images are generated in a synthetic way, so the best way to obtain more reliable metrics is to validate and test the model with totally realistic images.

#### 4.1.2. RGB Mask Detection

To reach the best precision model, the four YOLOv5 models were evaluated in an iterative way: firstly, an Evolve technique was used to find the best hiperperameters, and secondly, the obtained values in the final training were utilized (i.e., E#->E#.1). Table 5 shows the evaluations performed. The values of the finetune hyperparameters used in tests E1, E2, E3, and E4 are predefined by the authors [11], obtained after performing a medium model training of 50 epochs on the COCO dataset. To compare with our YOLOv5 family approach, we tested the face mask detection method, presented in [45], with our test dataset [44], to compare the obtained metrics of both models. This method uses an SSD framework, and it was trained on the dataset presented in [32]. To increase the speed of the network, the authors used a lite backbone with only 8 convolutional layers. Like our YOLOv5 models, the goal of this method is to detect faces and determine if they are wearing masks.

**Figure 9:**
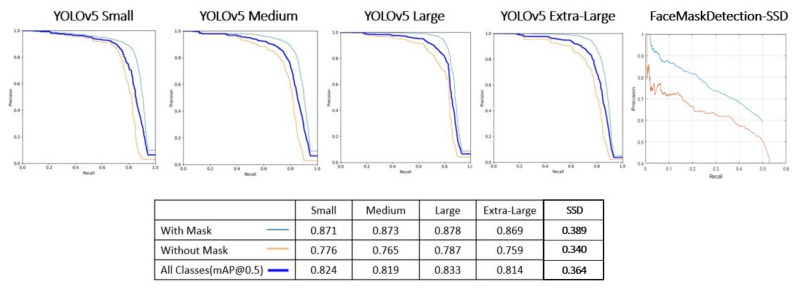
Presentation of Precision-Recall curves of models. In legend of each model, you can see average accuracies obtained for each class.

Table 6 presents the metrics obtained from performing the trials presented in Table 5, based on the hyperparameters obtained by the “Evolve” method (see Table 7). The average accuracies of each class can be calculated based on the analysis of the precision-recall curve, presented in Figure 9 for each of the models (YOLOv5 and FaceMaskDetection-SSD).

### 4.2. IR Detection

#### 4.2.1. Dataset

For the IR detection evaluations, MoLa IR CovSurv dataset was used. Table 8 presents the training, validation, and test subsets that form the final dataset used for training the mask and glasses object detection algorithms.

For the keypoint detection algorithms associated with the human caruncle, the dataset used in this task is constituted by a 70% fraction of the MoLa IR CovSurv dataset, used for mask and glasses detection, presented in Table 8, from which only the samples that present visible caruncles were selected, regardless of the presence or not of mask. Table 9 describes the subsets of this same dataset.

#### 4.2.2. Mask and Glasses Detection

As in Section 4.1.2, for the detection of masks and glasses in IR images, the four YOLOv5 models were evaluated in an iterative methodology. Table 10 describes the different trials performed to obtain the models used in the mask and goggles detection component. For tests E5, E6, E7, and E8, the hyperparameters presented by the authors [11] were used.

**Figure 10:**
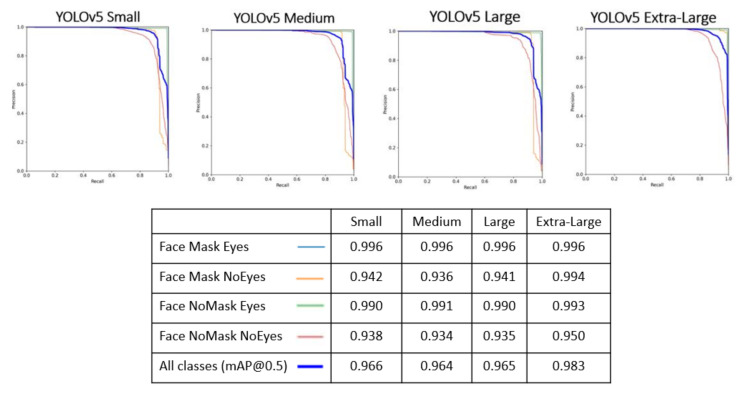
Presentation of Precision-Recall curves of models. In the legend of each model, you can see average accuracies obtained for each class.

Table 11 presents the metrics obtained from performing the tests presented in Table 10, based on the hyperparameters obtained by the “Evolve” method (see Table 12). The average accuracies of each class can be calculated based on the analysis of the precision-recall curve, presented in Figure 10 for each of the models (YOLOv5).

#### 4.2.3. Caruncle Detection

Table 13 shows the different tests performed for the task of detecting the area of the caruncle area of each eye. For the same model, 6 backbones were evaluated to select the highest performing and lowest computational requirements model.

Table 14 shows the results of the tests of Table 13. Normalized Mean Error (NME) is associated to the average error of the distance between the estimated points and the ground-truth points previously labeled, relative to the training samples. The “Inference Time” column refers to the time that each algorithm needs to analyze an image from the test dataset, presented in seconds. The column “Precision” refers to the accuracy of the algorithm on the test dataset within a margin of 5 pixels; that is, if the distance between the facial points calculated by the algorithm and the previously labeled facial points (groundtruth) is smaller than 5 pixels, a correct prediction is considered. Its calculation is given by the ratio between predictions considered true positives and all predicted positives.

## 5. Discussion

**Figure 11:**
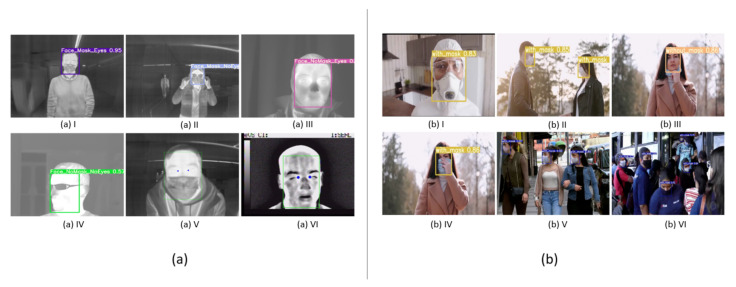
(**a-I - a-VI**) Examples of results obtained in images, from inference of selected algorithms for mask and glasses detection components and detection of zone corresponding to human caruncle. (**b-I - b-VI**) Examples of results obtained in images, from inference of selected YOLOv5 algorithm for mask detection.

In Section 4.1, the object detection algorithms, YOLOv5 family and FaceMaskDetection-SSD, are evaluated ore precisely to detect the presence or absence of masking. Although all the algorithms of the YOLOv5 family presented good results, the method to be used for the mask detection task is the Small model of the YOLOv5 architecture. This choice is justified by the fact that the different metrics obtained do not change substantially, since more layers were added along the remaining deeper models, and the task does not present a high degree of complexity since it is intended to detect only two distinct classes (with or without mask). Considering the inference times obtained are: 0.032 s for the Small model, 0.045 s for the Medium model, 0.062 for the Large model, and 0.089 s for the Extra-Large model. Thus, the best choice was to select the lightest model (Small), with 82.38% of mAP_0.5. Figure 11b shows qualitative results obtained on different samples, based on the inference of the selected model. The FaceMaskDetection-SSD method shows a 36.4% of mAP_0.5 when inferred on our test dataset. This may be because the model was trained on 7971 samples, which is a significantly lower number than our dataset. Hence, its inference capability on our test dataset is much lower. Furthermore, the FaceMaskDetection-SSD model has a lower complexity than our lighter model, YOLOv5s, with 1.01 M and 1.9 M parameters, respectively. Section 4.2 presents models capable of detecting the facial points of interest (using a thermographic camera) to be able to carry out effective temperature measurements as a way to screen for the potential presence of the SARS-CoV2 virus. This task is composed of two distinct steps: in the first step, and given that temperature measurements are not possible with the presence of glasses, object detection algorithms capable of detecting not only the presence of this object, but also the presence of masks were implemented (Section 4.2.2). In the second step, for the glasses and mask detection component, the algorithms forming the YOLOv5 architecture were selected, while for the face points detection component (Section 4.2.3), algorithms whose Backbones are made up of CNNs that are part of the Resnet and HrNetv2 architectures were selected. The results obtained by the different algorithms for both steps are quite satisfactory in the sense that these results experience practically no improvement with the use of deeper algorithms, since the number of classes and face points to be identified is quite low, in conjunction with the use of a highly uniform dataset whose samples are quite similar. Since the goal was to achieve high precision and low computational requirements, this led to the choice of the Small model for the glasses and mask detection aspect (corresponding to E5.1, with a precision of 81.86% and an inference time equal to the selected model for mask detection, 0.032 s), and the model with Backbone Resnet-50 (corresponding to E9, with a precision of 78.68% and an inference time of 0.024 s). Figure 11a shows qualitative results from the inference of the algorithms chosen for both tasks.

## 6. Conclusions

This article presents a system capable of detecting behaviors and risk factors of people within the scope of the COVID-19 pandemic, and more specifically, the implementation of algorithms for the detection of masks in public spaces, as well as the punctual execution of temperature measurements for the detection of possible cases of fever. Initially, a search was carried out associated with the existing state-of-the-art algorithms suitable for performing the proposed tasks. The selected algorithms belong to the themes of object detection and Keypoint Detection. The first task was mask detection in RGB images. As a basis for training the selected algorithms in this component, it was necessary to create a dataset and generate the respective labels. Regarding the dataset and given that the number of existing samples in this area is still scarce, a tool capable of applying synthetic masks to RGB images was developed, using pretrained models capable of locating the faces present and their respective facial points. Based on this information, a mask is subsequently applied within the existing types and textures to the facial points where it should be placed. The labels associated with this dataset were automatically sourced from the pretrained models used. Subsequently, using this dataset, multiple algorithms based on the YOLOv5 architecture were evaluated. After the training and respective evaluation of the results obtained, all models obtained good results, however, the Small model was the selected one (with a precision of 71.01%). This choice is justified because the obtained metrics are very similar despite the use of different and deeper models, mainly due to the fact that the required degree of complexity is not high because it is only intended to detect two different classes. Another reason is the balance between precision and real-time performance of the Small model regarding the other tested models.

For the temperature measurement component, it was also necessary to create a dataset consisting of thermographic images and generate the respective labels. In this case, algorithms were implemented both for mask and goggles detection, and for the detection of facial points associated with the human caruncle area, where the temperature measurement is performed with greater accuracy. The labels were originated in a semiautomatic way, i.e., based on the pretrained models enunciated in the previous task, as well as from manual labeling, image by image. For the mask and glasses detection task, the models coming from YOLOv5 architecture, associated with the object detection theme, were also tested, while for the face points detection task, algorithms were implemented, associated with the keypoint detection theme, which differ from each other in the present Backbone and whose constitutions correspond to variations of CNNs Resnet and HRNetv2. Respectively, the YOLOv5 Small algorithm was chosen (with a precision of 81.86%) as well as the algorithm whose Backbone is formed by the Resnet-50 architecture (with a precision of 78.68%). These choices, like the mask detection component, were based on the commitment between the obtained metrics and the real-time performance.

## Figures and Tables

**Table 1:** Datasets where proposed tool was applied.

Dataset	Description
Celeba ([28])	More than 200,000 images of faces. Images of just one person.
Coco ([29])	More than 320,000 images and more than 91 different objects, widely used for object detection tasks. We only used images where people are present in the most varied environments and contexts.
Helen ([30])	It consists of 2330 images of one or several people.
IMM ([31])	Consisting of 240 single-face images of 40 different people.
Wider ([32])	Over 32,000 images with different levels of scale and occlusion.
Group Images ([33])	More than 5000 images of groups of people.

**Table 2:** Thermographic datasets collected for training of mask, goggles, and caruncle zone detection algorithms.

Dataset	Description
IIITD In and Beyond Visible Spectrum Disguise Database ([37])	Thermographic images of 75 different people, with and without glasses, with and without mask.
UL-FMTV Database ([38])	Consisting of 18,210 thermographic images of samples of people with and without glasses.
Terravic Facial Infrared Database ([39])	It consists of 22,784 thermographic images of 18 different people.
IRIS Database ([40])	Consisting of thermographic images of 31 different people, with different lighting, expressions, and pose conditions.

**Table 3:** Classes to be identified and their IDs, in the masks and goggles detection task, in the thermography component.

Classe	ID
Face_Mask_Eyes	0
Face_Mask_NoEyes	1
Face_NoMask_Eyes	2
Face_NoMask_NoEyes	3

**Table 4:** Description of each subset of dataset used to detect presence or absence of masks.

Subset of Dataset	Description
Training	Images generated from the datasets presented in the Table 1. Consisting of a total of 40,972 samples.
Validation	It consists of 758 real samples of people with and without mask. The dataset used is presented in [43].
Test	It consists of 3441 real samples of people with and without mask. The dataset used is presented in [44].

**Table 5:** Tests performed using four object detection algorithms selected for mask detection task.

Trial	Dataset	Modelo	Epochs	Evolve	Evolve-Epochs	Hyperparameters
E1	30% Train, 30% Valid	YOLOv5s	3	Yes	100	FineTuneParams
E1.1	100% Train, 100% Valid	YOLOv5s	25	No	0	E1_EvolveParams
E2	30% Train, 30% Valid	YOLOv5m	3	Yes	100	FineTuneParams
E2.1	100% Train, 100% Valid	YOLOv5m	25	No	0	E2_EvolveParams
E3	30% Train, 30% Valid	YOLOv5l	3	Yes	100	FineTuneParams
E3.1	100% Train, 100% Valid	YOLOv5l	25	Não	0	E3_EvolveParams
E4	30% Train, 30% Valid	YOLOv5x	3	Yes	100	FineTuneParams
E4.1	100% Train, 100% Valid	YOLOv5x	25	No	0	E4_EvolveParams

**Table 6:** Metrics obtained from trials in Table 5. Bold lines represent results for the selected model.

	YOLOv5			
**Hyperparameter**	**Small (E1.1)**	**Medium (E2.1)**	**Large (E3.1)**	**Extra-Large (E4.1)**
**Precision**	**71.01%**	73.34%	82.77%	66.18%
**mAP_0.5**	**82.38%**	81.92%	83.25%	81.43%
**mAP_0.5:0.95**	**46.52%**	46.17%	45.96%	45.05%
**Recall**	**81.98%**	81.19%	77.68%	81.67%

**Table 7:** Values assigned to main hyperparameters after performing Evolve technique on YOLOv5 models for trials in Table 5.

	YOLOv5			
**Hyperparameter**	**Small (E5)**	**Medium (E6)**	**Large (E7)**	**Extra-Large (E8)**
**lr0**	0.0107	0.0049	0.00438	0.00417
**lrf**	0.24	0.17	0.0657	0.139
**Momentum**	0.98	0.98	0.98	0.924
**Weight_Decay**	0.00044	0.00031	0.00047	0.0004
**Box**	0.0291	0.0209	0.0224	0.0779
**Cls**	0.222	0.243	0.2	0.403

**Table 8:** Description of each subset of dataset used for mask and goggle detection in a thermographic context.

Subset of Dataset	Description
Train	12,254 samples
Validation	3501 samples
Test	1750 samples

**Table 9:** Description of each subset of dataset used for detection of the human caruncle area in thermographic context.

Subset of Dataset	Description
Train	8602 samples
Validation	2456 samples
Test	1229 samples

**Table 10:** Tests performed for four object detection algorithms selected for masks and glasses detection task using thermographic dataset.

Trial	Dataset	Model	Epochs	Evolve	Evolve-Epochs	Hyperparameters
E5	100% Train, 100% Valid	YOLOv5s	3	Yes	100	FineTuneParams
E5.1	100% Train, 100% Valid	YOLOv5s	20	No	0	E5_EvolveParams
E6	100% Train, 100% Valid	YOLOv5m	3	Yes	100	FineTuneParams
E6.1	100% Train, 100% Valid	YOLOv5m	20	No	0	E6_EvolveParams
E7	100% Train, 100% Valid	YOLOv5l	3	Yes	100	FineTuneParams
E7.1	100% Train, 100% Valid	YOLOv5l	20	No	0	E7_EvolveParams
E8	100% Train, 100% Valid	YOLOv5x	3	Yes	100	FineTuneParams
E8.1	100% Train, 100% Valid	YOLOv5x	20	No	0	E8_EvolveParams

**Table 11:** Metrics obtained from trials in Table 10. Bold lines represent results for selected model.

	YOLOv5			
**Metrics**	**Small (E5.1)**	**Medium (E6.1)**	**Large (E7.1)**	**Extra-Large (E8.1)**
**Precision**	**81.86%**	83.15%	85.23%	91.19%
**mAP_0.5**	**96.65%**	96.43%	96.53%	98.34%
**mAP_0.5:0.95**	**71.33%**	72.85%	72.60%	75.99%
**Recall**	**96.39%**	95.76%	96.78%	97.97%

**Table 12:** Values assigned to main hyperparameters after performing Evolve technique of the YOLOv5 models, for the trials in Table 10.

	YOLOv5			
**Hyperparameter**	**Small (E5)**	**Medium (E6)**	**Large (E7)**	**Extra-Large (E8)**
**lr0**	0.0033	0.00334	0.00376	0.00424
**lrf**	0.114	0.1	0.121	0.141
**Momentum**	0.972	0.98	0.968	0.959
**Weight_Decay**	0.00037	0.00031	0.00028	0.00026
**Box**	0.0385	0.237	0.0269	0.0273
**Cls**	0.299	0.265	0.214	0.2

**Table 13:** Trials performed for different algorithms selected for human caruncle detection task.

Trial	Backbone	Epochs	Learning Rate
E9	Resnet-50	5	0.002
E10	Resnet-101	5	0.002
E11	Resnet-152	5	0.002
E12	HrNetv2_w18	5	0.002
E13	HrNetv2_w32	5	0.002
E14	HrNetv2_w48	5	0.002

**Table 14:** Results obtained for each trial performed in human caruncle detection task. Bold lines represent best selected results for each model.

Trial	NME	Inference Time (seconds)	Precision	Average Error (pixels)
**E9**	**0.0945**	**0.024**	**78.68%**	**3.89**
E10	0.1012	0.04	78.44%	3.86
E11	0.1029	0.057	77.46%	3.92
E12	0.0905	0.107	82.1%	3.35
E13	0.0975	0.103	79.78%	3.77
E14	0.0971	0.104	79.05%	3.66

## Data Availability

Two datasets [2,3] were published and are available.
